# 2-Amino-4-(4-fluoro­phen­yl)-6-(naphthalen-1-yl)pyridine-3-carbonitrile

**DOI:** 10.1107/S1600536810050804

**Published:** 2010-12-08

**Authors:** Jian-Qiang Wang, Shi-Gui Tang, Cheng Guo

**Affiliations:** a College of Science, Nanjing University of Technology, Nanjing 210009, People’s Republic of China; b Biotechnology and Pharmaceutical Engineering, Nanjing University of Technology, Nanjing 210009, People’s Republic of China

## Abstract

The title compound, C_22_H_14_FN_3_, was prepared by a one-pot condensation using malononitrile, an aromatic aldehyde, a methyl ketone and ammonium acetate as reacta­nts under microwave irradiation. The pyridine ring is twisted with respect to the benzene ring and the naphthalene ring system, making dihedral angles of 41.9 (1) and 45.2 (1)°, respectively. In the crystal, mol­ecules are connected *via* inter­molecular N—H⋯N and C—H⋯F hydrogen bonds, forming a three-dimensional network.

## Related literature

For the use of 2-amino-3-cyano­pyridines as inter­mediates in the preparation of heterocyclic compounds, see: Shishoo *et al.* (1983 ▶). For bond-length data, see: Allen *et al.* (1987 ▶).
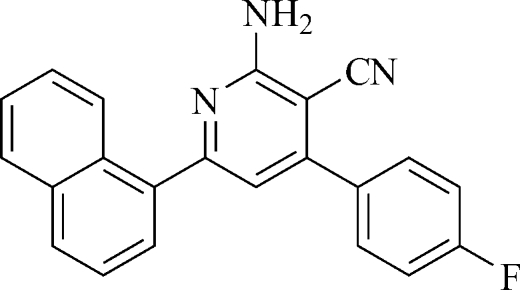


## Experimental

### 

#### Crystal data

C_22_H_14_FN_3_
*M* *_r_* = 339.36
Monoclinic, 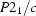

*a* = 14.531 (3) Å
*b* = 8.0900 (16) Å
*c* = 15.516 (3) Å
β = 110.04 (3)°
*V* = 1713.6 (6) Å^3^
*Z* = 4
Mo *K*α radiation
μ = 0.09 mm^−1^
*T* = 292 K
0.30 × 0.20 × 0.10 mm

#### Data collection

Enraf–Nonius CAD-4 diffractometer
Absorption correction: ψ scan (North *et al.*, 1968 ▶) *T*_min_ = 0.974, *T*_max_ = 0.991
3274 measured reflections
3144 independent reflections
2046 reflections with *I* > 2σ(*I*)
*R*_int_ = 0.056
3 standard reflections every 200 reflections  intensity decay: 1%

#### Refinement

*R*[*F* ^2^ > 2σ(*F* ^2^)] = 0.062
*wR*(*F* ^2^) = 0.177
*S* = 1.00
3144 reflections
236 parameters
H-atom parameters constrained
Δρ_max_ = 0.20 e Å^−3^
Δρ_min_ = −0.29 e Å^−3^

### 

Data collection: *CAD-4 Software* (Enraf–Nonius, 1985 ▶); cell refinement: *CAD-4 Software*; data reduction: *XCAD4* (Harms & Wocadlo,1995 ▶); program(s) used to solve structure: *SHELXS97* (Sheldrick, 2008 ▶); program(s) used to refine structure: *SHELXL97* (Sheldrick, 2008 ▶); molecular graphics: *SHELXTL* (Sheldrick, 2008 ▶); software used to prepare material for publication: *SHELXTL*.

## Supplementary Material

Crystal structure: contains datablocks I, global. DOI: 10.1107/S1600536810050804/vm2063sup1.cif

Structure factors: contains datablocks I. DOI: 10.1107/S1600536810050804/vm2063Isup2.hkl

Additional supplementary materials:  crystallographic information; 3D view; checkCIF report

## Figures and Tables

**Table 1:** Hydrogen-bond geometry (Å, °)

*D*—H⋯*A*	*D*—H	H⋯*A*	*D*⋯*A*	*D*—H⋯*A*
N2—H2*A*⋯N1^i^	0.86	2.33	3.149 (3)	158
C20—H20*A*⋯F^ii^	0.93	2.50	3.284 (4)	142

Symmetry codes: (i) 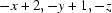; (ii) 
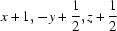
.

**Fig. 1.**
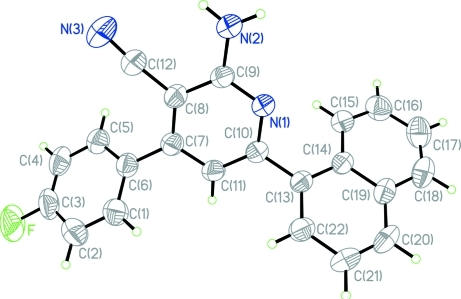
The molecular structure of (I), with the atom-numbering scheme and displacement ellipsoids at the 30% probability level.

**Fig. 2.**
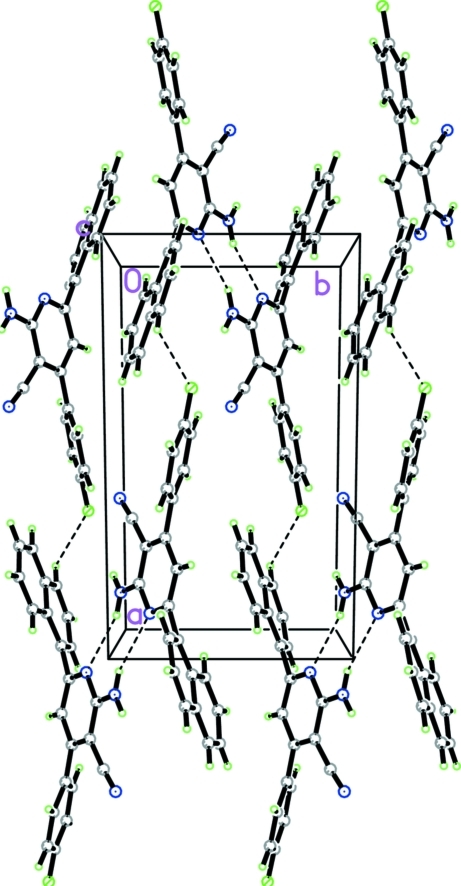
A packing diagram of (I). Intermolecular hydrogen bonds are shown as dashed lines.

## supplementary crystallographic information

### Comment

Derivatives of 2-amino-3-cyanopyridine are important and useful intermediates in preparing a varity of heterocyclic compounds (Shishoo *et al.*, 1983). Therefore, the synthesis of 2-amino-3-cyanopyridine derivatives attracts much interest in organic chemistry. Here, the single-crystal structure of the title compound (I), C_22_H_14_FN_3_, is reported.

The title compound (Fig. 1) consists of a pyridine ring bearing a 4-fluoro phenyl at C7, a naphthyl at C10, an amino group at C9, and a cyano group at C8. The pyridine ring is twisted with respect to the benzene ring and naphthalene ring, with dihedral angles of 41.9 (1)° and 45.2 (1)°, respectively. The bond lengths and angles are within the reported values (Allen *et al.*, 1987). Intermolecular N—H···N and C—H···F hydrogen bonds (Table 1, Fig. 2), and C—H···π and π–π stacking interactions help to stabilize the crystal structure.

### Experimental

The title compound (I) was prepared from a mixture of 4-fluorobenzaldehyde (2 mmol), malononitrile (2 mmol), 1-naphthaldehyde (2 mmol) and ammonium acetate (16 mmol) under microwave irradiation (6 min, WF-4000M microwave reaction system). After cooling to room temperature, the resulting solid product was filtered off and recrystallized from methanol to give compound (I). Colorless single crystals suitable for X-ray analysis were obtained by dissolving (I) (0.5 g) in methanol (20 ml) and slowly evaporating the solvent at room temperature for a period of about two weeks.

### Refinement

H atoms were positioned geometrically, with C—H = 0.93 Å and N—H = 0.86 Å for aromatic and amino H atoms, and constrained to ride on their parent atoms,with *U*_iso_(H) = *1.2U*_eq_(C/N).

### Crystal data

**Table:**

C_22_H_14_FN_3_	*F*(000) = 704
*M**_r_* = 339.36	*D*_x_ = 1.315 Mg m^−^^3^
Monoclinic, *P*2_1_/*c*	Melting point: 422.15 K
Hall symbol: -P 2ybc	Mo *K*α radiation, λ = 0.71073 Å
*a* = 14.531 (3) Å	Cell parameters from 25 reflections
*b* = 8.0900 (16) Å	θ = 9–13°
*c* = 15.516 (3) Å	µ = 0.09 mm^−^^1^
β = 110.04 (3)°	*T* = 292 K
*V* = 1713.6 (6) Å^3^	Block, colourless
*Z* = 4	0.30 × 0.20 × 0.10 mm

### Data collection

**Table:**

Enraf–Nonius CAD-4 diffractometer	2046 reflections with *I* > 2σ(*I*)
Radiation source: fine-focus sealed tube	*R*_int_ = 0.056
graphite	θ_max_ = 25.4°, θ_min_ = 1.5°
ω/2θ scans	*h* = 0→17
Absorption correction: ψ scan (North *et al.*, 1968)	*k* = 0→9
*T*_min_ = 0.974, *T*_max_ = 0.991	*l* = −18→17
3274 measured reflections	3 standard reflections every 200 reflections
3144 independent reflections	intensity decay: 1%

### Refinement

**Table:**

Refinement on *F*^2^	Secondary atom site location: difference Fourier map
Least-squares matrix: full	Hydrogen site location: inferred from neighbouring sites
*R*[*F*^2^ > 2σ(*F*^2^)] = 0.062	H-atom parameters constrained
*wR*(*F*^2^) = 0.177	*w* = 1/[σ^2^(*F*_o_^2^) + (0.1*P*)^2^ + 0.2*P*] where *P* = (*F*_o_^2^ + 2*F*_c_^2^)/3
*S* = 1.00	(Δ/σ)_max_ < 0.001
3144 reflections	Δρ_max_ = 0.20 e Å^−^^3^
236 parameters	Δρ_min_ = −0.29 e Å^−^^3^
0 restraints	Extinction correction: *SHELXL97* (Sheldrick, 2008), Fc^*^=kFc[1+0.001xFc^2^λ^3^/sin(2θ)]^-1/4^
Primary atom site location: structure-invariant direct methods	Extinction coefficient: 0.016 (3)

### Special details

**Table:**

Geometry. All e.s.d.'s (except the e.s.d. in the dihedral angle between two l.s. planes) are estimated using the full covariance matrix. The cell e.s.d.'s are taken into account individually in the estimation of e.s.d.'s in distances, angles and torsion angles; correlations between e.s.d.'s in cell parameters are only used when they are defined by crystal symmetry. An approximate (isotropic) treatment of cell e.s.d.'s is used for estimating e.s.d.'s involving l.s. planes.
Refinement. Refinement of *F*^2^ against ALL reflections. The weighted *R*-factor *wR* and goodness of fit *S* are based on *F*^2^, conventional *R*-factors *R* are based on *F*, with *F* set to zero for negative *F*^2^. The threshold expression of *F*^2^ > σ(*F*^2^) is used only for calculating *R*-factors(gt) *etc*. and is not relevant to the choice of reflections for refinement. *R*-factors based on *F*^2^ are statistically about twice as large as those based on *F*, and *R*- factors based on ALL data will be even larger.

### Fractional atomic coordinates and isotropic or equivalent isotropic displacement parameters (Å^2^)

**Table:**

	*x*	*y*	*z*	*U*_iso_*/*U*_eq_	
F	0.33760 (12)	0.1683 (3)	0.00030 (15)	0.1049 (8)	
N1	0.91754 (14)	0.3544 (2)	0.03593 (12)	0.0425 (5)	
C1	0.5928 (2)	0.2725 (4)	0.0869 (2)	0.0601 (8)	
H1B	0.6403	0.3032	0.1419	0.072*	
N2	0.87074 (16)	0.4912 (3)	−0.10104 (14)	0.0566 (6)	
H2A	0.9316	0.5106	−0.0915	0.068*	
H2B	0.8267	0.5267	−0.1502	0.068*	
C2	0.4983 (2)	0.2457 (4)	0.0839 (2)	0.0700 (9)	
H2C	0.4811	0.2591	0.1360	0.084*	
N3	0.6218 (2)	0.4965 (4)	−0.19941 (17)	0.0838 (9)	
C3	0.4303 (2)	0.1989 (4)	0.0021 (3)	0.0694 (9)	
C4	0.4519 (2)	0.1766 (4)	−0.0751 (2)	0.0673 (9)	
H4A	0.4039	0.1422	−0.1291	0.081*	
C5	0.54649 (19)	0.2056 (4)	−0.07242 (19)	0.0565 (7)	
H5A	0.5622	0.1927	−0.1253	0.068*	
C6	0.61841 (18)	0.2543 (3)	0.00910 (17)	0.0485 (6)	
C7	0.72116 (18)	0.2857 (3)	0.01511 (16)	0.0450 (6)	
C8	0.74512 (17)	0.3731 (3)	−0.05147 (15)	0.0453 (6)	
C9	0.84442 (17)	0.4055 (3)	−0.03910 (15)	0.0424 (6)	
C10	0.89413 (17)	0.2700 (3)	0.09979 (15)	0.0402 (6)	
C11	0.79866 (18)	0.2324 (3)	0.09168 (16)	0.0457 (6)	
H11A	0.7861	0.1716	0.1373	0.055*	
C12	0.6732 (2)	0.4389 (4)	−0.13286 (18)	0.0550 (7)	
C13	0.97766 (17)	0.2280 (3)	0.18524 (15)	0.0404 (6)	
C14	1.06861 (17)	0.1664 (3)	0.18259 (15)	0.0412 (6)	
C15	1.08360 (19)	0.1133 (3)	0.10156 (17)	0.0491 (6)	
H15A	1.0318	0.1182	0.0461	0.059*	
C16	1.1715 (2)	0.0555 (4)	0.1025 (2)	0.0650 (8)	
H16A	1.1792	0.0200	0.0484	0.078*	
C17	1.2509 (2)	0.0492 (4)	0.1853 (2)	0.0746 (9)	
H17A	1.3117	0.0130	0.1856	0.090*	
C18	1.2395 (2)	0.0954 (4)	0.2644 (2)	0.0667 (8)	
H18A	1.2928	0.0899	0.3187	0.080*	
C19	1.14842 (18)	0.1521 (3)	0.26713 (17)	0.0479 (6)	
C20	1.1338 (2)	0.1915 (4)	0.34944 (17)	0.0582 (8)	
H20A	1.1861	0.1836	0.4044	0.070*	
C21	1.0458 (2)	0.2407 (4)	0.35084 (17)	0.0586 (8)	
H21A	1.0369	0.2618	0.4064	0.070*	
C22	0.9674 (2)	0.2599 (3)	0.26778 (17)	0.0504 (7)	
H22A	0.9070	0.2952	0.2691	0.060*	

### Atomic displacement parameters (Å^2^)

**Table:**

	*U*^11^	*U*^22^	*U*^33^	*U*^12^	*U*^13^	*U*^23^
F	0.0381 (10)	0.142 (2)	0.1317 (18)	0.0019 (11)	0.0249 (11)	0.0234 (15)
N1	0.0410 (11)	0.0474 (12)	0.0378 (11)	0.0010 (9)	0.0116 (9)	0.0053 (9)
C1	0.0453 (16)	0.076 (2)	0.0558 (16)	−0.0088 (14)	0.0141 (13)	−0.0064 (15)
N2	0.0474 (12)	0.0720 (16)	0.0473 (12)	0.0008 (11)	0.0124 (10)	0.0190 (11)
C2	0.0548 (19)	0.089 (2)	0.072 (2)	0.0004 (17)	0.0296 (17)	−0.0007 (18)
N3	0.0727 (18)	0.107 (2)	0.0574 (15)	0.0126 (16)	0.0032 (13)	0.0246 (16)
C3	0.0340 (15)	0.081 (2)	0.091 (2)	0.0045 (14)	0.0193 (16)	0.0118 (19)
C4	0.0428 (16)	0.075 (2)	0.068 (2)	−0.0008 (15)	−0.0019 (14)	0.0053 (17)
C5	0.0471 (16)	0.0647 (18)	0.0512 (16)	−0.0013 (13)	0.0084 (13)	0.0002 (13)
C6	0.0408 (14)	0.0529 (16)	0.0486 (15)	−0.0005 (12)	0.0110 (12)	0.0025 (12)
C7	0.0433 (14)	0.0493 (15)	0.0402 (13)	−0.0029 (11)	0.0114 (11)	−0.0021 (11)
C8	0.0432 (14)	0.0498 (15)	0.0389 (13)	0.0013 (11)	0.0089 (11)	0.0012 (11)
C9	0.0451 (14)	0.0452 (14)	0.0361 (12)	0.0005 (11)	0.0129 (11)	0.0042 (11)
C10	0.0427 (14)	0.0390 (13)	0.0375 (12)	0.0005 (11)	0.0122 (11)	0.0017 (10)
C11	0.0453 (14)	0.0523 (15)	0.0393 (13)	−0.0046 (12)	0.0141 (11)	0.0053 (11)
C12	0.0497 (15)	0.0654 (18)	0.0464 (15)	0.0019 (14)	0.0119 (13)	0.0058 (14)
C13	0.0435 (13)	0.0395 (13)	0.0356 (12)	−0.0039 (11)	0.0101 (10)	0.0007 (10)
C14	0.0433 (14)	0.0362 (13)	0.0399 (13)	−0.0049 (11)	0.0088 (11)	0.0025 (11)
C15	0.0506 (15)	0.0503 (15)	0.0460 (14)	0.0005 (12)	0.0162 (12)	0.0008 (12)
C16	0.0658 (19)	0.0667 (19)	0.0694 (19)	0.0075 (16)	0.0320 (16)	−0.0025 (15)
C17	0.0518 (18)	0.079 (2)	0.091 (2)	0.0124 (16)	0.0209 (18)	−0.0018 (19)
C18	0.0486 (17)	0.067 (2)	0.069 (2)	0.0051 (15)	−0.0003 (14)	0.0018 (16)
C19	0.0459 (15)	0.0411 (14)	0.0477 (15)	−0.0033 (12)	0.0044 (11)	0.0028 (11)
C20	0.0617 (18)	0.0585 (17)	0.0378 (14)	−0.0053 (14)	−0.0044 (13)	−0.0006 (12)
C21	0.073 (2)	0.0647 (18)	0.0340 (14)	−0.0004 (15)	0.0122 (13)	−0.0050 (12)
C22	0.0535 (16)	0.0554 (16)	0.0408 (14)	0.0007 (13)	0.0142 (12)	−0.0001 (12)

### Geometric parameters (Å, °)

**Table:**

F—C3	1.360 (3)	C10—C11	1.383 (3)
N1—C10	1.340 (3)	C10—C13	1.498 (3)
N1—C9	1.344 (3)	C11—H11A	0.9300
C1—C2	1.376 (4)	C13—C22	1.364 (3)
C1—C6	1.387 (4)	C13—C14	1.426 (3)
C1—H1B	0.9300	C14—C15	1.414 (3)
N2—C9	1.342 (3)	C14—C19	1.427 (3)
N2—H2A	0.8600	C15—C16	1.356 (4)
N2—H2B	0.8600	C15—H15A	0.9300
C2—C3	1.369 (4)	C16—C17	1.403 (4)
C2—H2C	0.9300	C16—H16A	0.9300
N3—C12	1.145 (3)	C17—C18	1.347 (4)
C3—C4	1.350 (4)	C17—H17A	0.9300
C4—C5	1.381 (4)	C18—C19	1.414 (4)
C4—H4A	0.9300	C18—H18A	0.9300
C5—C6	1.393 (4)	C19—C20	1.401 (4)
C5—H5A	0.9300	C20—C21	1.347 (4)
C6—C7	1.486 (3)	C20—H20A	0.9300
C7—C8	1.391 (3)	C21—C22	1.406 (4)
C7—C11	1.396 (3)	C21—H21A	0.9300
C8—C9	1.413 (3)	C22—H22A	0.9300
C8—C12	1.438 (3)		
			
C10—N1—C9	118.1 (2)	C10—C11—C7	120.1 (2)
C2—C1—C6	121.1 (3)	C10—C11—H11A	120.0
C2—C1—H1B	119.4	C7—C11—H11A	120.0
C6—C1—H1B	119.4	N3—C12—C8	174.6 (3)
C9—N2—H2A	120.0	C22—C13—C14	119.6 (2)
C9—N2—H2B	120.0	C22—C13—C10	118.2 (2)
H2A—N2—H2B	120.0	C14—C13—C10	122.1 (2)
C3—C2—C1	118.0 (3)	C15—C14—C13	123.9 (2)
C3—C2—H2C	121.0	C15—C14—C19	117.9 (2)
C1—C2—H2C	121.0	C13—C14—C19	118.1 (2)
C4—C3—F	119.2 (3)	C16—C15—C14	121.8 (2)
C4—C3—C2	123.2 (3)	C16—C15—H15A	119.1
F—C3—C2	117.6 (3)	C14—C15—H15A	119.1
C3—C4—C5	118.8 (3)	C15—C16—C17	119.9 (3)
C3—C4—H4A	120.6	C15—C16—H16A	120.0
C5—C4—H4A	120.6	C17—C16—H16A	120.0
C4—C5—C6	120.3 (3)	C18—C17—C16	120.3 (3)
C4—C5—H5A	119.9	C18—C17—H17A	119.8
C6—C5—H5A	119.9	C16—C17—H17A	119.8
C1—C6—C5	118.6 (2)	C17—C18—C19	121.7 (3)
C1—C6—C7	119.4 (2)	C17—C18—H18A	119.1
C5—C6—C7	122.0 (2)	C19—C18—H18A	119.1
C8—C7—C11	117.0 (2)	C20—C19—C18	122.4 (2)
C8—C7—C6	122.8 (2)	C20—C19—C14	119.4 (2)
C11—C7—C6	120.1 (2)	C18—C19—C14	118.2 (2)
C7—C8—C9	119.8 (2)	C21—C20—C19	121.6 (2)
C7—C8—C12	123.3 (2)	C21—C20—H20A	119.2
C9—C8—C12	116.9 (2)	C19—C20—H20A	119.2
N2—C9—N1	116.4 (2)	C20—C21—C22	119.6 (2)
N2—C9—C8	121.8 (2)	C20—C21—H21A	120.2
N1—C9—C8	121.9 (2)	C22—C21—H21A	120.2
N1—C10—C11	123.1 (2)	C13—C22—C21	121.7 (3)
N1—C10—C13	115.9 (2)	C13—C22—H22A	119.2
C11—C10—C13	120.9 (2)	C21—C22—H22A	119.2
			
C6—C1—C2—C3	0.7 (5)	C8—C7—C11—C10	1.3 (4)
C1—C2—C3—C4	0.6 (5)	C6—C7—C11—C10	−176.3 (2)
C1—C2—C3—F	178.2 (3)	N1—C10—C13—C22	131.9 (3)
F—C3—C4—C5	−179.1 (3)	C11—C10—C13—C22	−43.9 (3)
C2—C3—C4—C5	−1.5 (5)	N1—C10—C13—C14	−44.0 (3)
C3—C4—C5—C6	1.1 (5)	C11—C10—C13—C14	140.2 (2)
C2—C1—C6—C5	−1.0 (4)	C22—C13—C14—C15	173.1 (2)
C2—C1—C6—C7	179.5 (3)	C10—C13—C14—C15	−11.0 (4)
C4—C5—C6—C1	0.1 (4)	C22—C13—C14—C19	−4.6 (3)
C4—C5—C6—C7	179.6 (3)	C10—C13—C14—C19	171.2 (2)
C1—C6—C7—C8	−137.1 (3)	C13—C14—C15—C16	−179.9 (2)
C5—C6—C7—C8	43.4 (4)	C19—C14—C15—C16	−2.1 (4)
C1—C6—C7—C11	40.4 (4)	C14—C15—C16—C17	−0.8 (4)
C5—C6—C7—C11	−139.2 (3)	C15—C16—C17—C18	2.1 (5)
C11—C7—C8—C9	−0.7 (4)	C16—C17—C18—C19	−0.3 (5)
C6—C7—C8—C9	176.9 (2)	C17—C18—C19—C20	176.2 (3)
C11—C7—C8—C12	−178.5 (2)	C17—C18—C19—C14	−2.6 (4)
C6—C7—C8—C12	−1.0 (4)	C15—C14—C19—C20	−175.1 (2)
C10—N1—C9—N2	179.3 (2)	C13—C14—C19—C20	2.8 (3)
C10—N1—C9—C8	0.3 (4)	C15—C14—C19—C18	3.8 (4)
C7—C8—C9—N2	−179.1 (2)	C13—C14—C19—C18	−178.3 (2)
C12—C8—C9—N2	−1.1 (4)	C18—C19—C20—C21	−178.0 (3)
C7—C8—C9—N1	−0.2 (4)	C14—C19—C20—C21	0.8 (4)
C12—C8—C9—N1	177.9 (2)	C19—C20—C21—C22	−2.7 (4)
C9—N1—C10—C11	0.5 (4)	C14—C13—C22—C21	2.9 (4)
C9—N1—C10—C13	−175.3 (2)	C10—C13—C22—C21	−173.1 (2)
N1—C10—C11—C7	−1.3 (4)	C20—C21—C22—C13	0.8 (4)
C13—C10—C11—C7	174.2 (2)		

### Hydrogen-bond geometry (Å, °)

**Table:**

*D*—H···*A*	*D*—H	H···*A*	*D*···*A*	*D*—H···*A*
C15—H15A···N1	0.93	2.50	2.997 (3)	114
N2—H2A···N1^i^	0.86	2.33	3.149 (3)	158
C20—H20A···F^ii^	0.93	2.50	3.284 (4)	142

Symmetry codes: (i) −*x*+2, −*y*+1, −*z*; (ii) *x*+1, −*y*+1/2, *z*+1/2.
